# The role of telemedicine in joint replacement surgery? An updated review

**DOI:** 10.1186/s42836-023-00196-1

**Published:** 2023-08-04

**Authors:** Ka Yau Li, Ping Keung Chan, Shun Shing Yeung, Amy Cheung, Wai Kwan Vincent Chan, Michelle Hilda Luk, Man Hong Cheung, Henry Fu, Kwong Yuen Chiu

**Affiliations:** 1 Li Ka Shing Faculty of Medicine, The University of Hong Kong, Hong Kong SAR, China; 2 Department of Orthopaedics and Traumatology, School of Clinical Medicine, The University of Hong Kong, Hong Kong SAR, China; 3 Physiotherapy Department, MacLehose Medical Rehabilitation Centre, Hong Kong SAR, China; 4 Department of Orthopaedics and Traumatology, Division of Joint Replacement Surgery, Queen Mary Hospital, Hong Kong SAR, China

**Keywords:** Telemedicine, Coronavirus disease 2019, Total joint arthroplasty, Prehabilitation

## Abstract

The usage of telemedicine and telehealth services has grown tremendously and has become increasingly relevant and essential. Technological advancements in current telehealth services have supported its use as a viable alternative tool to conduct visits for consultations, follow-up, and rehabilitation in total joint arthroplasty. Such technology has been widely implemented, particularly during the coronavirus 2019 (COVID-19) pandemic, to deliver postoperative rehabilitation among patients receiving total joint arthroplasty (TJA), further demonstrating its feasibility with a lower cost yet comparable clinical outcomes when compared with traditional care. There remains ample potential to utilize telemedicine for prehabilitation to optimize the preoperative status and postoperative outcomes of patients with osteoarthritis. In this review, various implementations of telemedicine within total joint arthroplasty and future application of telemedicine to deliver tele-prehabilitation in TJA are discussed.

## Introduction

Osteoarthritis is a common chronic disease in the elderly, causing pain, swelling, limited joint mobility stiffness and decreased quality of life due to inflammation of the synovial joint [1]. According to the World Health Organization, osteoarthritis is one of the four most prevalent chronic musculoskeletal conditions [2]. A study in Hong Kong showed that 7% of men and 13% of women had knee osteoarthritis (OA) [3]. With the older adults population in the Hong Kong increasing from 18.4% in 2019 to 33.3% in 2039, the disease burden of Hong Kong is substantial [4]. Early hip and knee OA are mostly treated with conservative management while total hip arthroplasty (THA) and total knee arthroplasty (TKA) have been proven a successful treatment for end-stage disease [5].

The availability of joint arthroplasty is frequently hampered by long waiting lists and prolonged waiting time may result in deterioration of patient’s preoperative pain and functional status [6]. The availability was particularly influenced by the situation of coronavirus disease 2019 (COVID-19) outbreak, during which hospitals worldwide reallocated resources to combat the challenges of this pandemic, sometimes by postponing elective surgeries. A local study reported a 53% and 54% reduction in the volume of elective arthroplasty operations and hospitalizations, respectively, compared with the data from the pre-COVID-19 pandemic period [7]. The orthopaedic clinic attendances also decreased by 20% [7]. All these contribute to an unexpected and accumulative rise in waiting time for elective arthroplasty during the COVID breakout.

Therefore, it is of particular importance to optimize the long waiting time at the period of a pandemic to intervene to, at least, maintain patients’ preoperative status and for better surgical outcomes and patient management and satisfaction. Preoperative function, such as performance in 6-min walk test and quadriceps strength, has been demonstrated to be a strong predictor for good postoperative short- and long-term outcomes in patients undergoing total joint arthroplasty [8, 9]. Interventions to improve preoperative function, therefore, are a sound approach to improve postoperative outcomes of TJA. However, the evidence from systematic reviews showed that prehabilitation before TJA is not conclusive. Moyer et al. (2017) [10] reported small-to-moderate improvements that varied with joints while Cabilan et al. (2016) [11] found no strong evidence to support prehabilitation’s effectiveness in improving post-TJA outcomes. A recent systematic review and meta-analysis involving 48 trials has demonstrated a moderate-certainty evidence favoring prehabilitation in terms of improving the function, muscle strength and flexion range in patients undergoing patients undergoing TKA, and muscle strength, abduction range and 36-item Short Form Health Survey score in patients with THA, respectively [12]. The variation in outcomes of prehabilitation could be attributed to several factors, importantly, adherence and exercise regimen design [13]. It is suggested that the intensity of prehab exercises should be a progressive and moderate-intensity program with a frequency of 4 times per week for a minimum of 3 months [13, 14]. However, the feasibility of such regime is questionable in situations where there is low accessibility to healthcare, for example, during the COVID-19 pandemic due to altered healthcare-seeking behaviors, public health orders and social restrictions [15, 16]. Telecommunication technologies and tele-healthcare services may offer a solution. Tele-prehabilitation provides patients with the flexibility and convenience to attend medical consultation and incorporate rehabilitation into their daily routine and clinicians with approaches to deliver interventions or to monitor patients’ progress and adherence to exercise protocol to improve pre-TJA functions [17, 18]. Therefore, in this article, the possible role of tele-prehabilitation in TJA is explored via discussing how tele-rehabilitation is implemented in post TJA and the evidence and feasibility of tele-prehabilitation in TJA.

## Methods

### Databases and search strategy

The medical literature was searched using PubMed, MEDLINE, EMBASE and Cochrane databases for published articles using the following terms: i.e., preoperative, perioperative, postoperative, telemedicine, telecommunication, telerehab, tele-rehabilitation, teleprehab, tele-prehabilitation, remote, virtual, internet-based, web-based, knee surgery, knee arthroplasty, knee replacement, total knee arthroplasty, total knee replacement, TKA, TKR, unicompartmental knee arthroplasty, UKA, hip surgery, hip arthroplasty, hip replacement, total hip arthroplasty, total hip replacement, THA and THR. Only publications in English were included. Full-text articles and abstracts from peer-reviewed journals only were eligible for inclusion.

## Current technologies in TJA telerehabiliation

Telemedicine refers to the exchange of medical information through electronic communication methods to improve patient health [19]. Clinician-to-clinician, clinician-to-patient, and patient-to-mobile health technologies are the three main forms of telemedicine contacts that ensure continuity of care [19]. Telemedicine between healthcare practitioners can be utilized for consultation with specialists and colleagues through video, email, and/or telephone and can be supported by the transfer of imaging data or clinical results for review. Patients and healthcare providers can use telemedicine to manage chronic illnesses, evaluate drug response, analyze patient-reported outcome measures (PROMs) and changes, evaluate mental health status, and provide routine follow-up care [19]. In the case of TJA, various technologies for telemedicine have been investigated to improve post-surgical outcomes.

### Integrated telerehabilitation system

The research on telerehabilitation systems has been rapidly expanding. In general, an integrated telerehabilitation system consists of a display, motion-tracking cameras or sensors, visual cameras and an application interface. The telerehabilitation application is loaded with personalized clinician-prescribed exercise protocols and can be displayed as an animated image to the patients at any time. Motion-tracking cameras or wearable motion sensors are the unique part of the integrated system allowing for detection of real-time joint motions and providing real-time feedback or artificial-intelligence (AI)-generated instructions to inform or improve patients’ performance of the exercise. Visual cameras record videos of the patients while they are performing the rehabilitation exercises, and clinicians can retrieve them to study patients’ performance. The application also allows for reporting of exercise outcomes and concerns raised by the patient, modification of exercise protocol, virtual meeting and monitoring of progress and patient-reported outcomes.

In a prospective study [20], Virtual Exercise Rehabilitation Assistant (VERA, Reflexion Health, San Diego, CA, USA), the telerehabilitation system being trialed by USA Food and Drug Administration (FDA), was shown to be a feasible and safe tool with good compliance of post-TKA or -UKA patients on rehabilitation. Pruv Bettger et al. (2020) [20] conducted an RCT comparing the use of VERA vs. in-person physiotherapy in 304 TKA patients and found that knee range of motion and gait speed were similar between groups at the 6th week after TKA and the telerehabilitation group demonstrated higher exercise compliance (88.3% vs. 65.4%; *P* < 0.001), lower costs at the 12th week after discharge (median of USD$1,050 versus USD$2,805; *P* < 0.001) and no difference in PROMs as compared with the in-person group. Similar efficacy has also been shown in another study using other integrated telerehabilitation systems, reporting significantly better outcomes in telerehabilitation group compared with traditional face-to-face rehabilitation: Timed Up and Go test (TUG) 4.87 s less at the 6th month (*P* < 0.001) and higher KOOS at the 3rd month (*P* < 0.001) and at the 6th month (*P* = 0.006) [21].

### Remote coaching

Remote coaching usually involves delivering home exercise protocols to patients in the form of instructional videos via websites, applications and so on. The performance of the patient may be recorded and reviewed by the therapist during regular videoconference. Another common format would be an exercise session supervised by a therapist via real-time visual feedback and verbal cues by using video call and other telecommunication methods. Expensive equipment, such as motion sensors and a sophisticated application system, is not needed yet remote coaching may cost as much as installation of an Integrated telerehabilitation system considering the salary of a therapist [22]. Bini and Mahajan (2017) [23] reported that the utilization of hospital-based resources was 60% less in the remote coaching group for TKA patients than in the traditional physiotherapy group and no significant difference was found in any clinical outcome and satisfaction with care between the two groups. Promising results have also been demonstrated in recent studies. In an RCT of 70 THA, Nelson et al. (2020) [24] found no difference between the groups in the sub-scores of the life of quality of the Hip Disability and Osteoarthritis Outcome Score (HOOS), strength and balance, but, interestingly, a significantly higher satisfaction (*P* = 0.001) and compliance (*P* = 0.048) in the telerehab group. Wijnen et al. (2020) [25] also found a similar result that the group following remote coaching in THA, when compared with the historical control group of usual care after THA, achieved faster TUG at the 12th week and the 6th month after THA (*P* = 0.02) and scored significantly better on the HOOS sub-domain of sport and recreational activities (*P* = 0.04 at the 4th week, 12th week; *P* = 0.03 at the 6th month), hip-related QoL at the 6th month (*P* = 0.02), better on SF-36 subdomain physical role limitations at the 12th week (*P* = 0.04) and the 6th month (*P* = 0.03).

### Web- or application-based materials

Web- or App-based telerehabilitation usually consists of an interactive e-learning online platform or an application containing videos about daily exercise programs and learning materials about surgery and common post-surgical concerns. The content of the platform will be adjusted remotely according to the patient’s progress. A patient care team can review patients’ progress and address any concerns raised by the patients. The advantage of such telerehabilitation methods is that they demand less resources. Zachwieja et al. (2020) [26] found that patients who used out-patient physiotherapy services alone for post-TKA rehabilitation incurred a cost of USD$1,444 per person and USD$100 for the web-based only services. However, there is currently a lack of RCT comparing the effectiveness of web-based material vs. traditional face-to-face rehabilitation (rehab) in TJA. In a retrospective study of 941 THA patients, 295 (31.3%) of the patients originally enrolled in web-based-rehab-only group were eventually prescribed out-patient therapy service at the 4th week, among whom 88.2% due to perceived need, 10.8% due to patient request and 1% due to inability to use the web-based platform [27]. Interestingly, female sex and a lower preoperative SF-12 physical component but not age and home environment were associated with an increased need for face-to-face therapy service if only web-based rehabilitation materials were given. Klement et al. (2019) [27] concluded that web-based rehabilitation platforms may not benefit all patients undergoing arthroplasty.

### Wearable physical activity sensors

Commercially available wearable activity sensors are feasible for passively tracking patient activities, such as step counts, distance walked, and caloric measures of exercise intensity in TJA. The information will be stored and feedbacked to the patient via a mobile device such as a smartphone. Such incentive feedback to increase physical activity was demonstrated in a recent randomized controlled trial (RCT) involving 97 patients undergoing TJA, showing that patients wearing activity sensors walked for 28 min longer on postoperative day 1 compared with patients who received standard physical therapy alone [28]. Another recent RCT reported that TJA subjects who received activity feedback had significantly higher activity levels but not patient-reported outcome measures, including EuroQol-5 score and KOOS, after TJA over 6 weeks and 6 months, compared with subjects who did not receive feedback [29]. Moreover, it was found that, in obese patients undergoing TKA, the fitness tracker failed to increase overall compliance with the exercise program [30]. Therefore, a wearable activity sensor is an adjunct to post-TJA rehabilitation for altering daily steps and activity time only but it may not lead to better post-TJA outcomes and higher adherence to a rehabilitation program. Patterson et al. (2020) [31] further investigated the use of wearable activity sensors for stratifying TJA patients to identify sedentary patients who could benefit from extra physical therapy and found that the sedentary group in the early postoperative period reported clinically significantly greater pain improvement at the 6th week compared with the group which quickly restore to or exceeded their pre-injury level of activity. Hence, the prescription of wearable sensors should expand from the concept of a feedback device for daily steps to a device for monitoring of daily activity level, in which sedentary group should warrant further attention to their progress of recovery after TJA.

### Automated mobile phone messaging

An automated Mobile Messaging Protocol was developed by Day et al. (2018) [32] for post-TJA care. It would automatically send mobile phone messages to patients daily from 1-week before operation to 2-week after operation. Messages to remind patients of the date of surgery, encourage patients to be active, to remind them to examine their skin, and information regarding nil per oral (NPO) status and medication instructions were included. Their result showed that patients receiving messages were more likely to have a good understanding of health responsibilities (*P* = 0.024) and feel that the care team demonstrated shared decision-making (*P* = 0.024) when compared with historical control.

## Evidence and feasibility of tele-prehabilitation in TJA

In recent years, there has been a rapidly growing number of clinical studies and evidence regarding the use of tele-rehabilitation for postoperative care of patients undergoing TJA. Systematic reviews reported that post-TJA tele-rehabilitation is less expensive and not inferior or even better than face-to-face physical therapy in terms of clinical outcomes and patient satisfaction, recommending tele-rehabilitation as a substitute for traditional rehabilitation and augmentation for postoperative care in TJA [33, 34]. Our review has also examined a variety of technologies used in tele-rehabilitation, including integrated tele-rehabilitation systems, remote coaching, web-based materials, wearable physical activity sensors and automated mobile phone messaging (Table 1). They all demonstrated their non-inferiority to face-to-face rehabilitation or, at least, their value as adjuncts to postoperative care in TJA. Besides, platforms, such as integrated telerehabilitation systems, remote coaching, and web-based materials, are proven to be safe and feasible tools to carry out exercise protocols for most elderly patients. Hence, it is certain that prehabilitation using telemedicine is also a feasible option and such technical and clinical success could be translated into the design of prehabilitation protocol for the benefit of patients waiting for TJA.

**Table 1:** Summary of current technologies in TJA telerehabilitation

	Integrated telerehabilitation system	Remote coaching	Web or application based materials	Wearable physical activity sensor	Automated mobile phone messaging
Method of delivering videos of exercise protocol and instruction	Purchased system application as part of the integrated telerehab system	Interactive e-learning websites, applications or any platforms convenient	Interactive e-learning websites, applications or any platforms convenient	Not for exercise prescription	Not for exercise prescription
Set-up requirement					
Pre-purchased application	+	+ ∕ −	+ ∕ −	−	−
Tracking cameras or sensors	+	−	−	−	−
Visual cameras	+	+	−	−	−
Real-time feedback	+ (by AI)	+ (by therapist)	−	−	−
Progress evaluation via virtual meeting	+	+	+	+ ∕ −	−
Approximated Cost (USD$)	> 1,000 [20]	> 1,000 [22]	100 [25]	70 [29]	Insignificant direct cost to already mobile user
Role in TJA telerehabilitation	As an alternatives to traditional face-to-face rehabilitation after TJA			Monitoring of daily activity level during post TJA rehabilitation	Improve understanding of health responsibilities

**Fig. 1:**
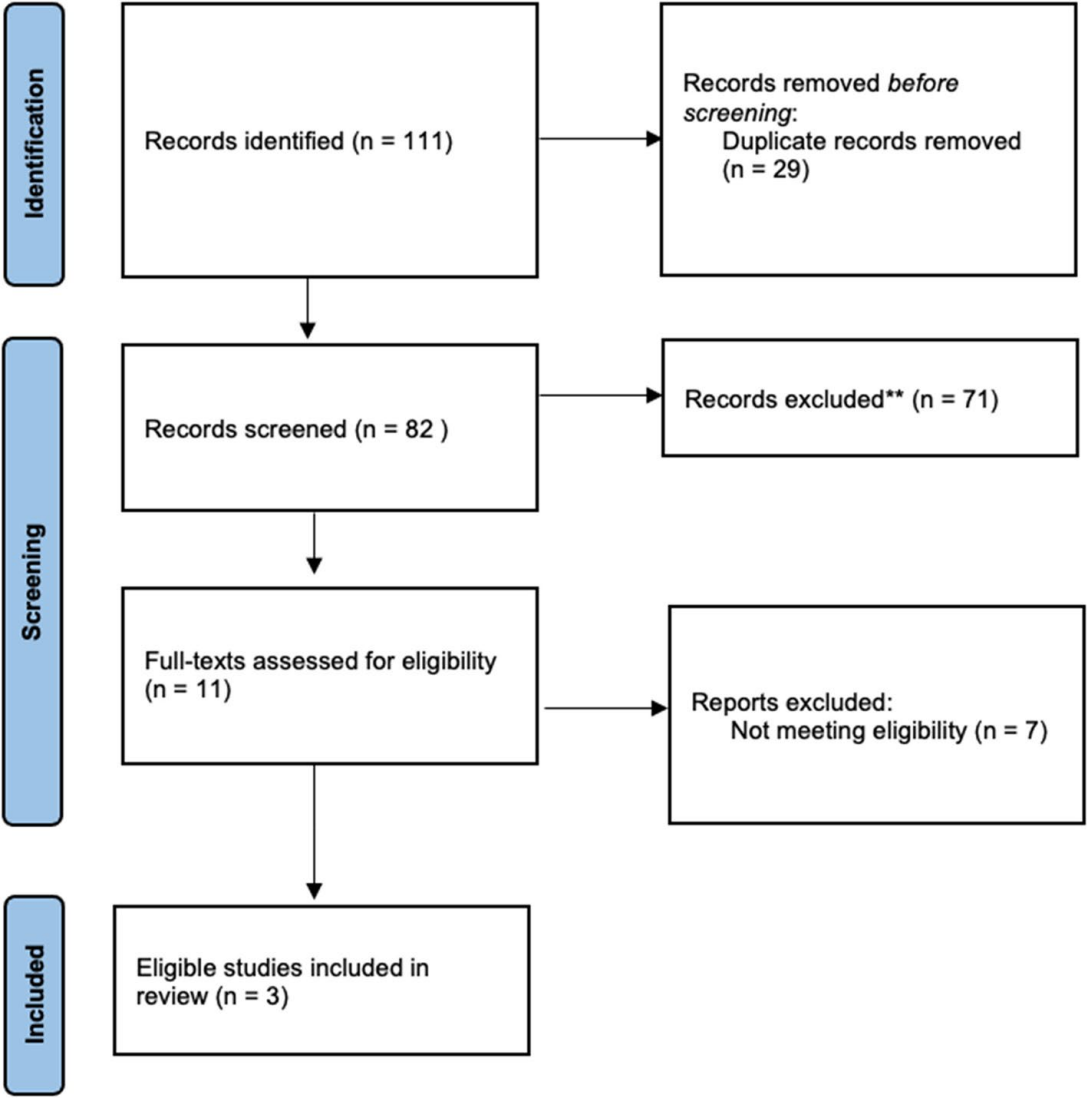
PRISMA flow diagram

However, the major hurdle to the implementation is the scarcity of evidence for tele-prehabilitation in TJA. Three studies investigating the use of tele-prehabilitation in TJA were identified after conducting a search in PubMed, MEDLINE, EMBASE, and Cochrane databases for published articles using the terms as aforementioned in the *Method* section (Fig. 1). Chughtai et al. (2019) [35] (*n* = 114) demonstrated that TKA patients receiving tele-prehabilitation had a shorter length of stay than the control group. Doiron-Cadrin et al. (2020) [36] conducted a three-arm parallel pilot RCT allocating a total of 34 TJA patients into three groups who were on (1) an in-person 12-week prehabilitation program; (2) a tele-prehabilitation program; (3) usual care with a single home visit and educational booklets only. The tele-prehabilitation was in form of remote coaching twice per week for 12 weeks. The prehabiliation protocol involved the exercise of range of motion of the hip and knee, strengthening of the hip and knee muscles, as well as proprioceptive exercises, cardiovascular warming-up, education regarding medication usage, and ice application for both tele-prehab and face-to-face prehab groups. The result of the RCT eventually did not demonstrate any significant difference in PROMs between the three groups due to limitations such as inadequate study sample size (*n* = 34). Another limitation is perhaps the inadequate intensity of the prehabilitation protocol. For the elderly, it is suggested that the intensity of prehabiltation exercises should be a progressive and moderate-intensity exercise program at a frequence of 4 times per week, lasting for a minimum of 3 months [14]. The RCT’s prehab protocol may not meet the suggested intensity. In contrast, a similarly designed three-arm parallel RCT by An et al. (2021) [37] reported significant differences in the time-by-group interaction, favoring the tele-rehabilitation group for 60/s extension peak, 180/s extension peak torque, TUG time WOMAC pain, WOMAC functional outcome, and WOMAC total score at 6 weeks post TKA. It was perhaps due to a large sample size of 60 TKA patients and, more importantly, a more intensive prehab protocol. The tele-rehabilitation was in the form of a bi-daily remote coaching with real-time visual feedback and verbal cues from a therapist. The prehab protocol was, to some extent, similar to the exercise prescribed in the study by Doiron-Cadrin et al. (2020) [36] but with higher intensity. Subjects in tele-prehab were asked to perform the prehab exercise for 30 min per session, 2 times per day, 5 days per week for 3 weeks before TKA while the patient education group was asked to follow the same protocol after a face-to-face education session while those in the control group received no prehabilitation. At the end of the study, the tele-rehabilitation group also perform better in aspects such as TUG, WOMAC pain, WOMAC functional outcome, and WOMAC total score than the patient education and control group.

The highlights from research about the tele-prehabilitation in TJA is/are the importance of the design of prehabilitation protocol, which has to be in the right intensity, right types of exercise and in the right platform of telemedicine. A short intensive course of tele-prehab involving progressive moderate exercise protocol has been shown to be beneficial to patients undergoing TKA [36]. It is worthwhile to investigate the outcome of a longer course of tele-prehab vs. a short-course one given the long waiting list of TJA. It is also uncertain what the minimum exercise dosage is or if any dose-dependent relationship between prehab exercise and improved post-TJA outcomes. This could be an area of future research.

**Fig. 2:**
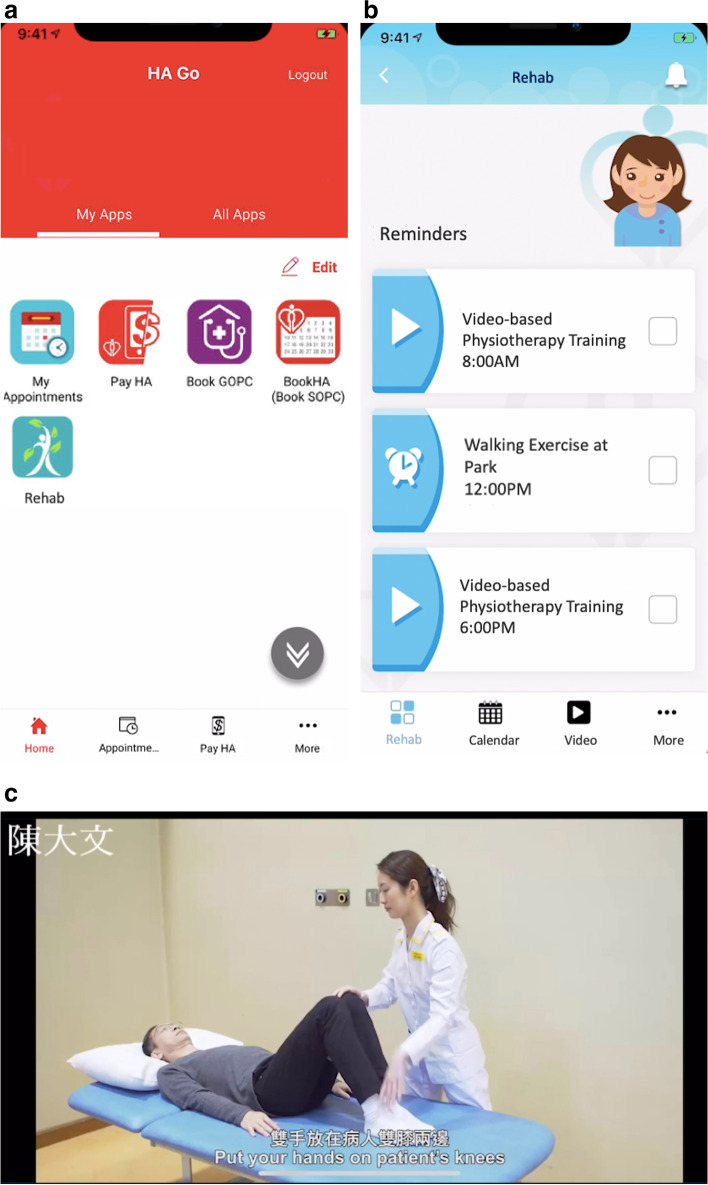
**a, b** HA GO application system for tele-rehabilitation by Hospital Authority Hong Kong [39]; **c** Attention notice, visual and text instructions and number of repetitions and set are shown for each exercise in the HA GO App [39]

Other factors involving patients and therapists should also be taken into account when telemedicine is implemented. Recent qualitative studies identified a number of challenges in the implementation of a tele-prehabilitation program [17, 18]. These challenges included patient learning curve to adapt to new technologies, interface not being patient-friendly, hearing and comprehension difficulties, interruptions during telemedicine consultations, as well as the lack of opportunity to meet and seek advice and support from patients with a similar background [17, 18]. Similar themes were also identified in our local program with tele-rehabilitation initiated in March 2020 during the COVID-19 pandemic involving twenty-one patients with knee osteoarthritis recruited from orthopaedic clinics [38]. The tele-rehabilitation consisted of online videos delivered via an application system (HA Go) (Fig. 2a, b) or via social media platforms to prescribe exercise and encourage home exercise with progression for osteoarthritis patients whose access to out-patient and day-patient services were affected during the COVID-19 pandemic [39]. All patients had eight sessions of tele-rehabilitation by telephone, each lasting 10 min, twice a week for four weeks. All of the patients were taught illness management and encouraged to exercise on a daily basis. The severity of pain, walking tolerance, difficulty with stairs, EuroQoL Index, and disease knowledge, patient satisfaction and overall improvement in the numeric global rating of change scale (NGRCS) were all evaluated (Table 2) and NPRS at rest, at movement, EQ5D3L Index showed statistically significant improvement after the telerehabilitation (*P* < 0.05). Our local experience showed that the tele-rehabilitation program was feasible with good acceptance from patients with knee osteoarthritis. However, patients' basic knowledge of disease management and knowledge of the progression of training were often inadequate. Besides, the majority of the senior patients used smartphones for calling only and the use of mobile application programs or video conferencing via social media platforms was often difficult for them. Thus, therapists in charge of the program must properly educate them, with encouragement, about the concept of a successful exercise program and their role and responsibility in the management of knee osteoarthritis. Therapists must also be aware of any potential technical difficulty that the patient may face when they attempt to initiate tele-rehabilitation services. A proper patient education session was also a key factor in the success of a prehab program.

**Table 2:** Result of tele-rehabilitation for patients with knee osteoarthritis during COVID-19 in our local program (*n* = 21)

	Data (mean ± standard deviation)	
Subject	21 (5 male and 16 female, mean age 67.5 ± 7.19)	
	Pre	Post
Upstairs difficulties (5 levels, moderate or above)	52%	33%
Downstairs difficulties(5 levels, moderate or above)	76%	57%
Walking Tolerance (mintues)	35.00 ± 28.94	40.24 ± 33.60
EQ5D3L index (1 is best)	0.74 ± 0.09	0.81 ± 0.11*
Knowledge(5 is total correct)	2.48 ± 1.81	4.24 ± 0.94*
NPRS		
At rest	2.19 ± 5.46	0.81 ± 2.68
At movement	4.10 ± 5.94	2.00 ± 3.74*
At walking	8.05 ± 4.36	6.86 ± 4.51
Patient satisfaction	4.33 ± 0.73	
NGRCS	2.95 ± 2.22	

*Abbreviations*: *EQ5D3L* EuroQOL-5 Dimension Three-level Version Questionnaire, *NPRS* Numerical Pain Rating Scale, *NGRCS* Numeric Global Rating of Change Scale

^*^P < 0.05

Finally, telemedicine undoubtedly provides a new platform for prehabilitation, with advantages such as lower cost, remote access to rehabilitation services at home at any time, easier monitoring of progress and decreased exposure to disease outbreaks in the community. Currently, there is no quantitative research comparing the adherence, satisfaction and clinical outcomes of various technologies used in tele-rehabilitation. Therefore, whether to use integrated tele-rehabilitation systems, remote coaching, web-based materials, wearable physical activity sensor and/or automated mobile phone messaging for patients waiting for TJA may depend on patients’ needs and accessibility, economic issues and clinicians’ assessment. Not all platforms for tele-prehabilitation are beneficial and can satisfy all patients and clinicians [27]. Clinicians should be vigilant when prescribing such services. It is important to review patients' progress and identify poor responders who may need other options of prehabilitation.

## Conclusion

With growing acceptance and evidence proving the effectiveness of telemedicine for postoperative care in total joint arthroplasty, a translation of such technical and clinical success into the design of prehabilitation protocol could benefit patients waiting for TJA. Platforms to conduct tele-prehabilitation such as integrated tele-rehabilitation system, remote coaching, wearable physical activity sensor, web or application-based materials, automated mobile phone messaging could be adopted as alternatives or adjuncts to traditional prehabilitation programs in future clinical practice. The main challenges for such adaptation include age-related accessibility and the scarcity of evidence evaluating tele-prehabilitation for TJA patients. Further research is required for effectiveness assessment and accessibility optimization of tele-prehabilitation for TJA patients.

## Data Availability

Not applicable.

## Abbreviations

COVID-19: Coronavirus Disease 2019
TJA: Total Joint Arthroplasty
OA: Osteoarthritis
TKA: Total Knee Arthroplasty
AI: Artificial Intelligence
VERA: Virtual Exercise Rehabilitation Assistant
FDA: USA Food and Drug Administration
RCT: Randomized Controlled Trial
PROMs: Patient-Reported Outcome Measures
TUG: Timed Up and Go Test
KOOS: Knee injury and Osteoarthritis Outcome Score
HOOS: Hip Disability and Osteoarthritis Outcome Score
QoL: Quality of Life
SF-36: 36-Item Short Form Survey
SF-12: 12-Item Short Form Survey
EuroQol-5: EuroQOL-5 Dimension Questionnaire
WOMAC: Western Ontario and McMaster Universities Osteoarthritis Index
EQ5D3L: EuroQOL-5 Dimension Three-level Version Questionnaire
NPRS: Numerical Pain Rating Scale
NGRCS: Numeric Global Rating of Change Scale
